# SlUPA-like, a bHLH Transcription Factor in Tomato (*Solanum lycopersicum*), Serves as the Crosstalk of GA, JA and BR

**DOI:** 10.3390/ijms252413419

**Published:** 2024-12-14

**Authors:** Pengyu Guo, Xin Cheng, Yunshu Wang, Guoping Chen, Xuqing Chen, Yingwu Yang, Xiuhai Zhang, Zongli Hu

**Affiliations:** 1 Laboratory of Molecular Biology of Tomato, Bioengineering College, Chongqing University, Chongqing 400044, China; guopengyu@stu.cqu.edu.cn (P.G.); chengxin599@163.com (X.C.); wangyunshu@cqu.edu.cn (Y.W.); chenguoping@cqu.edu.cn (G.C.); yangyinwu@cqu.edu.cn (Y.Y.); 2 Institute of Grassland, Flowers and Ecology, Beijing Academy of Agriculture and Forestry Sciences, Beijing 100097, China; chenxuqing@baafs.net.cn

**Keywords:** tomato (*Solanum lycopersicum*), *SlUPA-like*, plant hormone, *SlGID2*

## Abstract

The bHLH (basic Helix–Loop–Helix) transcription factor serves as pivotal controller in plant growth and development. In a previous study, the overexpression of *SlUPA-like* in *Solanum lycopersicum* L. Ailsa Craig (AC^++^) altered the JA (Jasmonic acid) response and endogenous GA (Gibberellic acid) content. However, the detailed regulation mechanism was not fully explored. In the present research, we found that the overexpression of *SlUPA-like* influenced the accumulation of GA, JA and BR (Brassinolide). RNA-Seq data illustrated that the expression levels of genes related to these plant hormones were significantly affected. Additionally, the interaction of SlUPA-like with SlMYB21, SlMYC2 and SlDELLA was characterized by employing Y2H (Yeast Two-Hybrid) and BiFC (Bimolecular Fluorescence Complementation) assay. Furthermore, Dual-LUC (Dual-Luciferase) assay and EMSA (Electrophoretic Mobility Shift Assay) identified that SlUPA-like directly targeted the E-box motif in the promoter of *SlGID2* and activated the transcription of *SlGID2*. These results shed light on the potential role of SlUPA-like in mediating crosstalk among multiple plant hormones and established a robust theoretical framework for further unraveling the functions of SlUPA-like transcription factors in the context of plant growth and hormone signal transduction.

## 1. Introduction

Transcription factors constitute a pivotal class of proteins that play a crucial role in the regulation of gene expression and signal transduction, plant growth and development, response to environment and other progresses. Among these, the bHLH (basic Helix–Loop–Helix) transcription factor family, widely distributed across eukaryotes, stands as the second-largest family following MYB in the plant kingdom [1,2]. The “bHLH” stems from the highly conserved domains within these proteins, characterized by a basic region and an HLH region. The basic region, situated at the N-terminus of the bHLH domain, consists of 17 amino acids, facilitating its binding to promoter DNA sequences. Conversely, the HLH region, positioned at the C-terminus, encompasses two amphipathic α-helices separated by a variable loop and plays a pivotal role in protein interactions [3]. Based on their ability to specifically bind to target gene promoters, bHLH transcription factors are categorized into two types: typical and atypical [4]. Notably, comprehensive genome-wide identification studies of bHLH transcription factors have been conducted in diverse species, including *Brassica napus* [5], potato (*Solanum tuberosum*) [6], pepper (*Capsicum annuum*) [7], cucumber (*Cucumis sativus*) [8], *Ginkgo biloba* [9], maize (*Zea mays*) [10], peach (*Amygdalus persica*) [11] and others.

To date, numerous pieces of evidence have robustly supported the pivotal role of bHLH transcription factors in various facets of plant growth and development, plant hormone signal transduction and abiotic stress responses, among others. For instances, the silencing of *PhFBH4* via VIGS (Virus-Induced Gene Silencing) resulted in a prolonged flower lifespan while the *PhFBH4*-overexpressed lines exhibited the opposite phenotype [12]. AtLP1 and AtLP2 served as the positive regulator in leaves’ longitudinal cell elongation determination by directly targeting the promoter of *AtLNG1* and *AtLNG2* in *Arabidopsis* [13]. OsBLR1, a bHLH transcription factor, positively controlled leaf angle and grain length via BR signaling transduction in rice [14]. SlbHLH95 participated in fruit ripening and the regulation of multiple metabolisms in tomato [15]. TabHLH49 positively empowered the drought tolerance of wheat by regulating the expression of *WZY2* [16]. In rice, the protein–protein interaction between OsbHLH61 and OsbHLH96 influenced the transcript level of genes in the salicylic acid signaling pathway, further governing the defense of rice against brown planthopper [17]. Induced by JA and targeted directly by MYC2, the knockout of *SlJIG* (a bHLH TF gene) decreased the tolerance of tomato to cotton bollworm and *B. cinerea* [18]. The rice bHLH073 functioned in the regulation of plant height, internode elongation and panicle exertion determination by monitoring the transcript level of *OsKO1* and *OsKO2*, which were involved in the gibberellin biosynthesis pathway [19]. 

In our previous research, we isolated the homologous gene of *Caupa20* (*Capsicum annuum*) in tomato and characterized its biological function by transforming it into wild type. The results illustrated that the overexpression of *SlUPA-like* induced cell enlargement and generated significant alteration to plant morphology. Moreover, the transgenic lines exhibited a decreased resistance to abiotic stress by affecting the ABA pathway. In addition, the overexpression of *SlUPA-like* altered the gibberellin content and jasmonic acid response [20]. The overexpression of *SlUPA-like* affected flowering time and enhanced the biosynthesis of ethylene [21], and response to exogenous IAA and ABA were also characterized [22]. 

However, the aforementioned study only described the phenotypes resulting from the overexpression of this gene using transgenic methods, without elucidating the regulatory mechanisms by which this gene participates in multiple hormones signaling pathways. In this study, we aimed to elucidate the function of SlUPA-like in multiple phytohormone crosstalk pathways, using molecular biology techniques combined with physiological and biochemical analyses. We found that the overexpression of *SlUPA-like* altered the content of endogenous gibberellin, jasmonic acid and brassinolide. Additionally, *SlUPA-like* physically interacted with SlMYB21, SlMYC2 and SlDELLA. Moreover, *SlUPA-like* bound to the promoter of *SlGID2*, further activating its transcript level. These results collectively indicated that *SlUPA-like* possibly functioned as a crosstalk in multiple plant hormone. Our findings provided insights into the role of this gene in tomato hormone responses, laying a theoretical foundation for the further functional characterization of *SlUPA-like*.

## 2. Results

### 2.1. Multiple Sequence Alignment and Cis-Acting Analyses of SlUPA-like Promoter

**Figure 1:**
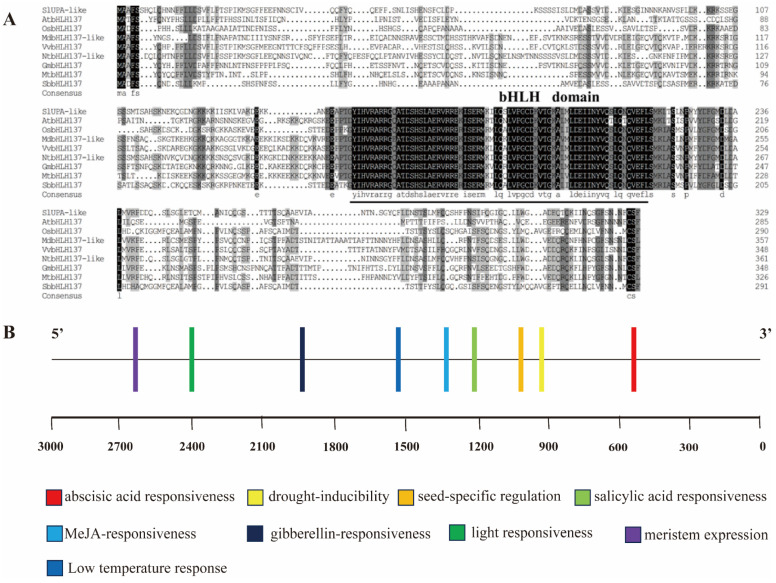
(**A**) The protein multiple sequence alignment of *SlUPA-like* and homologous genes from various plant species, including *At* (*Arabidopsis thaliana*, NP_568745), *Os* (*Oryza sativa*, NP_001409737), *Md* (*Malus domestica*, XP_028959633), *Vv* (*Vitis vinifera*, XP_002284464), *Nt* (*Nicotiana tabacum*, NP_001312696), *Gm* (*Glycine max*, XP_006578721), *Mt* (*Medicago truncatula*, XP_003630566), *Sb* (*Sorghum bicolor*, XP_002462650). (**B**) The prediction of *cis*-acting element based on the 3 kb sequence upstream of *SlUPA-like* in the SGN database, using online database PlantCare.

By employing the Protein Blast function in the NCBI database, we conducted a search for homologous genes of *SlUPA-like* in other species, subsequently retrieving their protein sequences respectively. The multiple sequence alignment, depicted in Figure 1A, revealed that the *SlUPA-like* transcription factor encompassed the characteristic bHLH domain. For exploring the regulation mechanism of *SlUPA-like*, we downloaded a 3 kb promoter sequence before the start code, analyzed the *cis*-acting elements using the online tools PlantCare and further visualized them. As shown in Figure 1B, the *cis*-acting elements involved in gibberellin, MeJA response, low-temperature response and drought inductility were identified, demonstrating that *SlUPA-like* possibly participated in plant hormone and abiotic stress response (The detailed information was shown in Table S1).

### 2.2. Overexpression of SlUPA-like Alters the Accumulation of Endogenous Gibberellin, Jasmonate and Brassinolide

**Figure 2:**
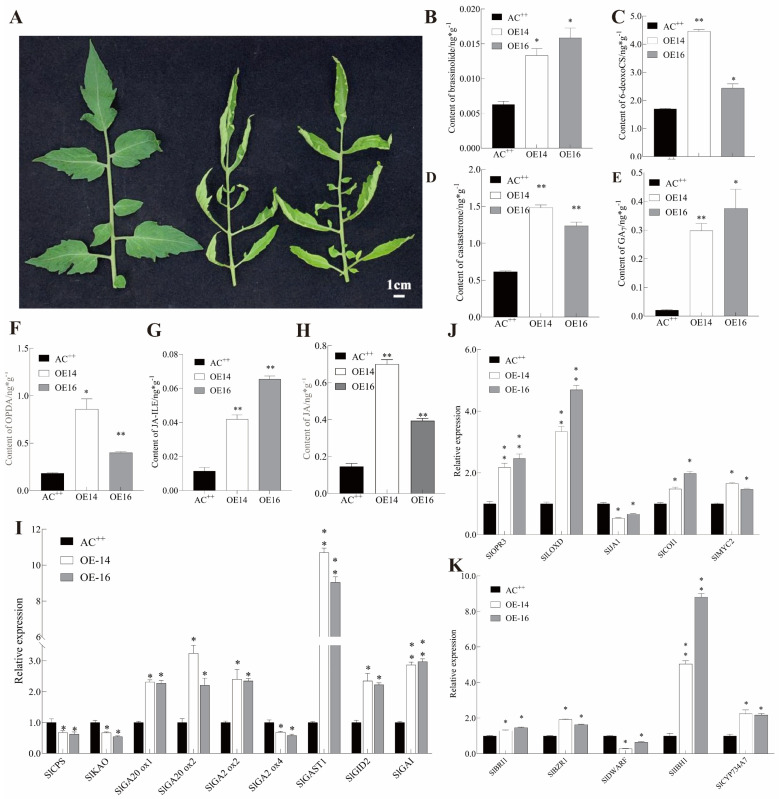
(**A**) The phenotype of leaves collected in the present study. (**B**–**H**) The measurement of plant hormone in wild-type and transgenic lines, (**B**) brassinolide; (**C**) 6-deoxocastasterone; (**D**) CS; (**E**) GA_7_; (**F**) JA; (**G**) OPDA; (**H**) JA-ILE. (**I**–**K**) The detection of transcript level of genes involved in GA ((**I**), *CPS*, *KAO*, *GA20ox1*, *GA20ox2*, *GA2ox2*, *GA2ox4*, *GAST1*, *GID2* and *GAI*), JA ((**J**), *OPR3*, *LOXD*, *JA1*, *COI1* and *MYC2*) and BR ((**K**), *BRI1*, *BZR1*, *DWERF*, *IBH1* and *CYP734A7*). Error bars represent the standard error of the mean (*n* = 3). (**) *p* < 0.01 and (*) *p* < 0.05 between the AC^++^ and transgenic plants by the *t* test.

For further exploring the function of *SlUPA-like*, we examined the expression levels in different parts of the overexpression lines to ensure the accuracy of the experiment (Figure S1). Upon collating the phenotypic data observed in the transgenic lines and the analysis of *cis*-acting elements within the promoter region of *SlUPA-like*, it became evident that it possibly played a significant role in various plant hormone pathways. To rigorously test our hypothesis, we assessed the content of endogenous gibberellin, jasmonate and brassinolide in both AC^++^ and *SlUPA-like*-overexpressed lines. As illustrated in Figure 2A, the leaf morphology of transgenic plants has been significantly changed compared with wild type; the leaves were collected to determine the profiles of these plant hormones in the transgenic lines and wild-type lines. Notably, the *SlUPA-like*-overexpressed lines displayed a remarkable increase in the content of brassinolide, 6-deoxocastasterone and castasterone compared to AC^++^ (Figure 2B–D). Similarly, the content of bioactive gibberellin GA_7_ (Figure 2E) also demonstrated an increase. Additionally, the content of JA, OPDA and JA-ILE increased significantly in the transgenic lines compared to the wild type (Figure 2F–H).

Subsequently, we performed qRT-PCR to track the expression levels of genes related to GA, BR and JA. The findings indicated that most of the detected genes related to gibberellin exhibited an evident induction, encompassing biosynthesis genes *GA20ox1* and *GA20ox2*, degradation genes like *GA2ox2* and signal transduction genes including *GAST1*, *GID2* and *DELAA*. In contrast, the transcript levels of *CPS*, *KAO* and *GA2ox4* were downregulated in the transgenic lines (Figure 2I). Similarly, genes involved in MeJA biosynthesis (*OPR3* and *LOXD*) and MeJA signal transduction (*COI1* and *MYC2*) exhibited upregulation in the *SlUPA-like*-overexpressed lines when compared with AC^++^ (Figure 2J). Moreover, *CYP734A7* (involved in BR biosynthesis), *BRI1*, *BZR1* (associated with BR signal transduction) and *IBH1* were upregulated, while *DWARF* (related to BR biosynthesis) was inhibited (Figure 2K). 

### 2.3. RNA-Seq Analysis of Young Leaves Between ov-SlUPA-like and AC^++^

**Figure 3:**
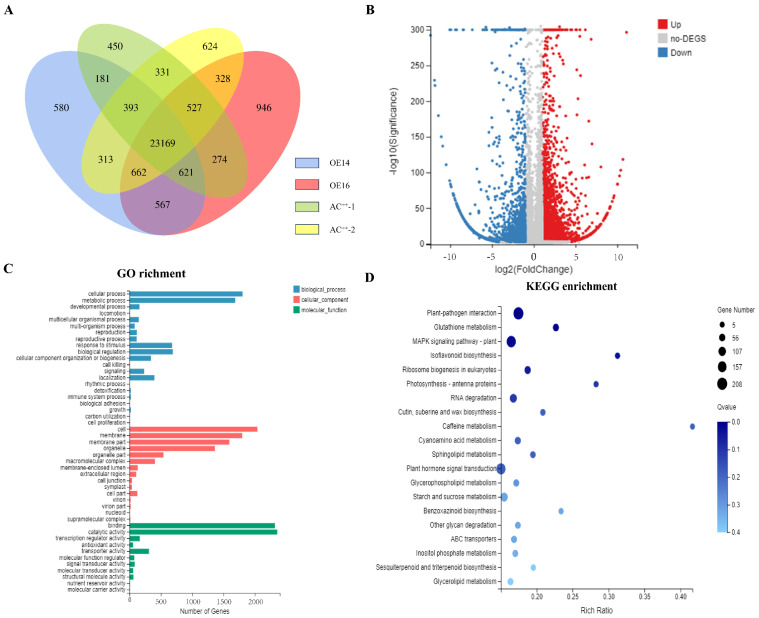
RNA-seq analyses of the mature leaves in both AC^++^ and *35S:SlUPA-like* lines. (**A**) A Venn diagram illustrating the overlap and unique expressed genes identified in the samples. (**B**) A volcano plot depicting the differentially expressed genes (DEGs) between the AC^++^ and *35S:SlUPA-like* lines, with 3404 upregulated and 2131 downregulated DEGs. (**C**) Gene Ontology (GO) analyses were performed on the DEGs to determine their functional categories. (**D**) Kyoto Encyclopedia of Genes and Genomes (KEGG) enrichment analysis was conducted to identify enriched pathways among the major upregulated and downregulated DEGs. The “Rich factor” indicates the ratio of DEGs associated with a specific KEGG pathway to the total number of DEGs.

To gain further insight into the molecular biological function of *SlUPA-like*, an RNA-seq strategy was employed to focus on the DEGs (differentially expressed genes) affected by the overexpression of *SlUPA-like*. In total, 25,339 genes were detected in both AC^++^ and OE samples. A total of 1147 genes were detected only in the AC^++^, while 2535 genes occurred only in the ov-*SlUPA-like* lines (Figure 3A). Moreover, 5535 DEGs were explored between the AC^++^ and ov-*SlUPA-like* lines, of which 3404 genes were upregulated and 2131 genes downregulated (Figure 3B). To categorize these DEGs, we conducted GO functional enrichment analysis, classifying them into three categories: biological process (BP), cellular component (CC) and molecular function (MF). Within the BP category, the DEGs were predominantly associated with metabolic and cellular processes. In terms of MF, the majority of DEGs were involved in catalytic activity. Within the CC category, the DEGs were primarily linked to cellular components and membranes (Figure 3C). Additionally, based on the KEGG pathway enrichment analysis, the differentially expressed genes were mainly enriched in pathways such as plant–pathogen interaction, the MAPK signaling pathway in plants, plant hormone signaling transduction, RNA transport and caffeine metabolism (Figure 3D).

### 2.4. Analysis of DEGs

**Figure 4:**
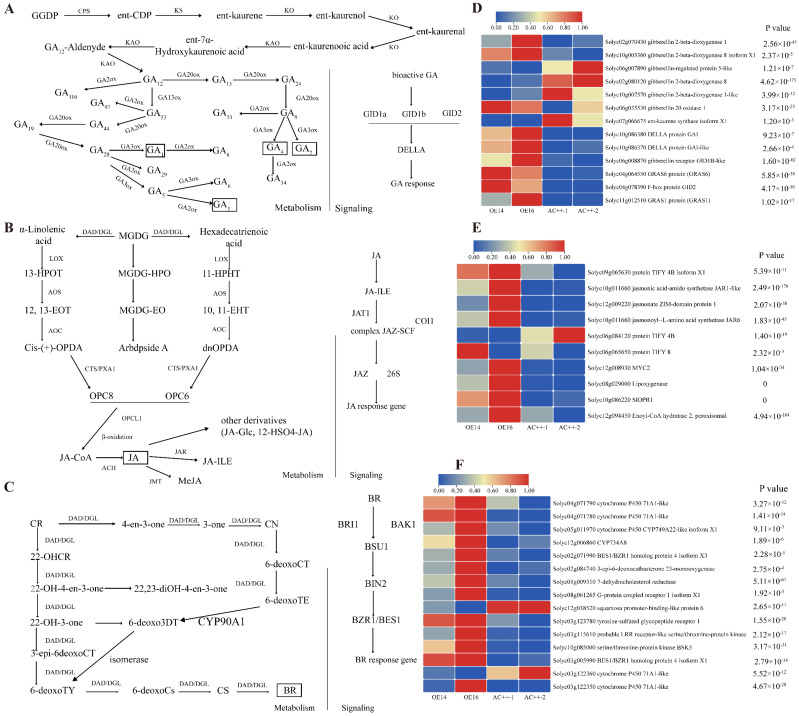
(**A**–**C**) Metabolic and signaling transduction network diagram of GA, JA and BR. (**D**–**F**) The visualization of DEGs involved in GA, JA and BR. The heatmap of DEGs based on RNA-Seq involved in JA (**A**), GA (**B**) and BR (**C**).

Building upon the characterization of the transgenic lines, we proceeded to analyze the RNA-seq data pertaining to plant hormone regulation, encompassing GA, JA and BR. Firstly, we mapped the metabolic and signaling pathways of GA, JA and BR (Figure 4A–C). Subsequently, we isolated the DEGs involved in GA, JA and BR in RNA-Seq data, Figure 4D–F. The results showed the transcript level of genes involved in the gibberellin pathway, *GA20ox1*, *DELLA*, *GID1B-like*, *GID2* and GRAS transcription factor genes *GRAS1*/*6* were up-regulated significantly in transgenic lines (Figure 4D). In addition, the mRNA abundance of *MYC2*, *OPR1*, *lipoxygenase*, *JAR6* and *JAR1-like* were apparently promoted in OE lines compared with AC^++^ (Figure 4E). Similarly, we also visualized the DEGs involved in the BR pathway (Figure 4F). To validate the accuracy of the RNA-Seq data, we employed qRT-PCR analysis and detected the mRNA abundance of six genes, namely *Solyc05g04629*, *Solyc04g082920*, *Solyc07g042830*, *Solyc03g005990*, *Solyc08g029000* and *Solyc04g064550*. The transcript levels of these genes exhibited significant enhancement in the transgenic lines compared to the wild type, providing a robust confirmation of the reliability of the RNA-Seq data (Figure S2).

### 2.5. Screening of Interacting Proteins of SlUPA-like

**Figure 5:**
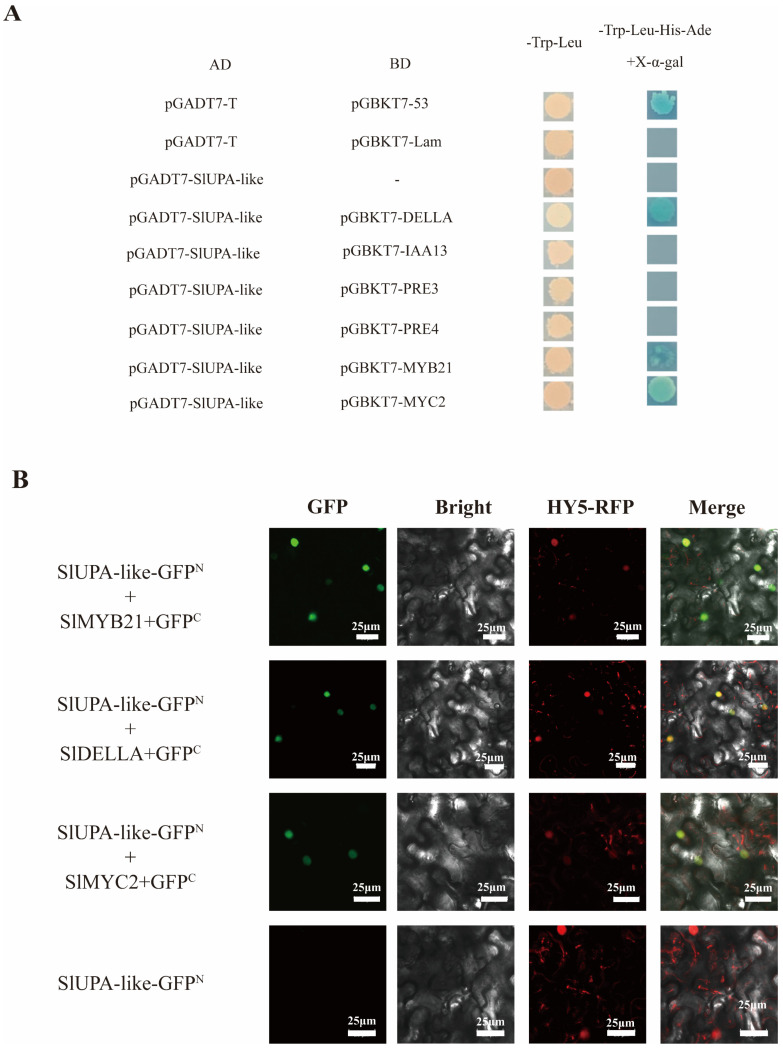
The protein-protein identification between SlUPA-like and MYB21, MYC2 and GAI through Y2H (**A**) and BiFC assay (**B**). Bar means 25 μm. In Y2H assay, pGADT7-T and pGBKT7-53 were set as the positive control, while pGADT7-T and pGBKT7-Lam were set as the negative control. BD refers to the DNA-binding domain, and AD represents the activation domain. *SlUPA-like* was cloned into pGADT7, and others were cloned into pGBKT7. The protein–protein interaction was identified using the synthetic defined double dropout (SD DDO) medium (**left**) and SD quadruple dropout (SD QDO) medium with X-α-gal(5-bromo-4-chloro-3-indolyl-α-d-galactopyranoside) (**right**). In BiFC assay, the HY5-RFP was set as nuclear localization signal. *SlUPA-like* was cloned into GFP^N^, while *GAI*, *MYB21* and *MYC2* were cloned into GFP^C^. The first column is the field of view under the green fluorescence signal, the second column is the bright field of view, the third column is the field of view under the red fluorescence signal, and the fourth column is the merged image of the three fields of view. The bar means 25 μm.

In general, bHLH transcription factors function in the regulation of plant growth and development, either independently or through interactions with other transcription factors [23,24]. Whether SlUPA-like participated in regulating plant hormone pathways by interacting with other transcription factors was a question which needed to be explored. Based on the RNA-Seq data, we selected *IAA13*, *PRE3*, *PRE4*, *DELLA*, *MYB21* and *MYC2* to perform Y2H. It has been identified that *PRE3* and *PRE4* located at the downstream of gibberellin and the overexpression of *SlUPA-like* induced the expression level of *PRE3/4* [20,25]. *MYC2* and *DELLA* were the crucial genes in the JA and GA pathways, respectively [26,27]. It has been identified that MYB21 functioned as a crosstalk between JA and GA [28]. For *IAA13*, it was illustrated that the overexpression of *SlUPA-like* altered the sensitivity to exogenous IAA, and *IAA13* was found to be the most upregulated gene among the auxin-related genes in RNA-seq data [22]. Initially, we cloned the CDS sequence of *SlUPA-like* into pGBKT7 and generated pGBKT7-*SlUPA-like*. When we performed Y2H assay, transactivation assay showed that SlUPA-like has a self-activation ability. To more accurately identify the protein–protein interaction between SlUPA-like and other TFs, we cloned *SlUPA-like* into pGADT7 while other genes including *PRE3*, *PRE4*, *DELLA*, *MYC2* and *MYB21* were cloned into pGBKT7. The results showed that PRE3, PRE4, DELLA, MYC2 and MYB21 did not have a self-activation ability and that SlUPA-like could interact with DELLA, MYC2 and MYB21. (Figure 5A). Further, BiFC assay was employed to confirm the interaction by transient expression in *Nicotiana benthamiana* epidermal cells, further confirming the protein interaction between SlUPA-like and SlDELLA/SlMYB21/SlMYC2 (Figure 5B).

### 2.6. SlUPA-like Directly Targets SlGID2

**Figure 6:**
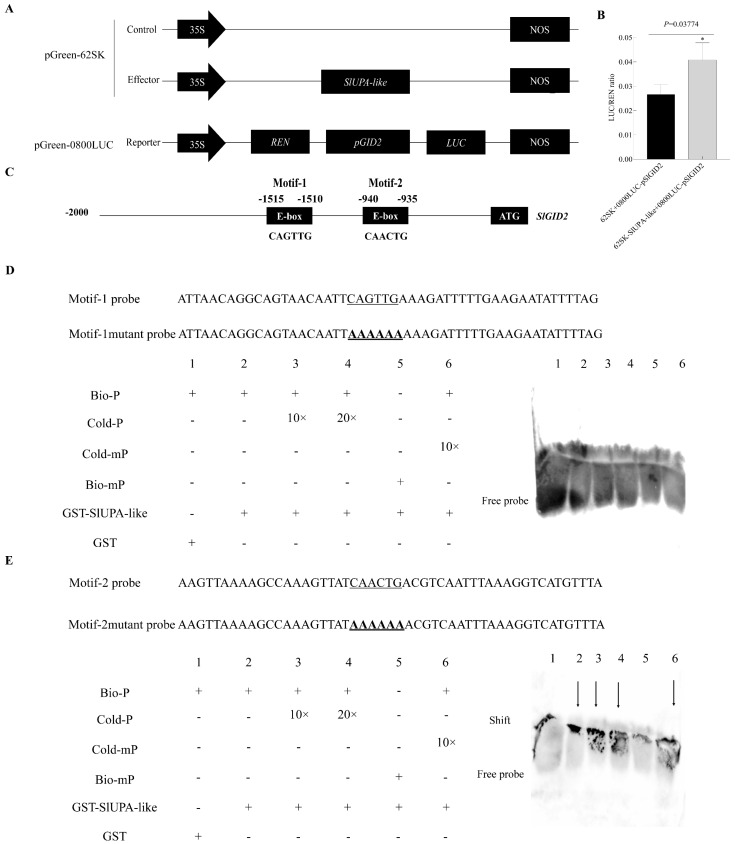
The regulation of *SlUPA-like* on *SlGID2*. (**A**) Schematic diagram of Dual-LUC assay. The empty pGreen-62SK was set as the control, and the pGreen-62SK-*SlUPA-like* was set as the effector. The 2 kb promoter sequence before ATG of *SlGID2* was cloned into pGreen 0800LUC to drive the LUC and 35S promoter drives REN as internal control. (**B**) The results of Dual-LUC showed that *SlUPA-like* dramatically activated the transcript of *SlGID2*. Error bars represent the standard error of the mean (*n* = 3). * means *p* < 0.05. (**C**) There were two E-box motifs in the 2 kb promoter sequence before ATG. (**D**,**E**) EMSA assay identified that *SlUPA-like* regulated *SlGID2* by binding to motif 2 in its promoter. The underlined sequences represent E-box elements, and the bold and underlined sequences represent mutated E-box elements. Bio-P and Bio-mP mean probe and mutant probe labeled by biotin, respectively. Cold-P and Cold-mP mean cold probe and mutant cold probe without biotin label, respectively. The image was acquired using the ChemiDocTM MP Image system from BIO-RAD, Image Lab Touch Software (version 3.0.1.14).

For further exploring the regulation net of SlUPA-like involved in plant hormones, we selected the four possible candidate downstream genes and explored their regulation relationship. By employing a Dual-LUC experiment, we found that *SlUPA-like* significantly activated the transcript of *SlGID2* (Figure 6A,B) only while there was no evident activation on other genes (Figure S3), including *SlOPR1*, *SlJAZ2* and *SlGID1-b*. Moreover, it was shown that there were two E-box motifs in 2kb promoter sequence. Further, we performed EMSA assay to determine which E-box motif could be bound by *SlUPA-like*. The results showed that SlUPA-like bound to motif 2 rather than motif 1, demonstrating that SlUPA-like governed the transcript of *SlGID2* by binding to the E-box motif CAACTG (Figure 6C–E).

## 3. Discussion

In the plant kingdom, bHLH transcription factors represent a significant class of proteins that play crucial roles in regulating plant growth and development, metabolic processes and environmental responses. For example, the silencing of *bHLH132* results in the abnormal growth and development of tomato plants and increases their susceptibility to infection by *X. euvesicatoria* [29]. SlbHLH92 could have induced the transcript level of *SlLCD1*, a gene involved in the biosynthesis of H2S, and the overexpression of *SlbHLH92* conferred an increased tolerance to salt stress [30]. It has been characterized that SlMS32 participated in the regulation of pollen and tapetum development [31]. The overexpression of *SlbHLH152* exhibited the tolerance to Fe deficiency and increased the accumulation of Fe. SlbHLH152 directly targeted *SlbHLH68* and functioned in the regulation of iron homeostasis [32]. The knockout of *Nrd1*, which was induced by immunity-inducing flgII-28 peptide, exhibited a promoted tolerance to *Pseudomonas syringae pv.* tomato (Pst) DC3000 [33]. The overexpression of *SlCES* showed the increased tolerance to chilling and had an effect on GA homeostasis [34]. The overexpression of *CubHLH1*(*Citrus unshiu* Marc.) in tomato generated a dwarf phenotype, inhibited the accumulation of lycopene and the altered the transcript level of genes involved in carotenoid biosynthesis [35]. These reports demonstrated that bHLH played a vital role in the various aspects of the plant.

In plant molecular biology research, systematic family gene analysis helps to better elucidate the biological functions of genes within a specific family, providing a theoretical foundation for practical applications in production. Previous reports systemically summarized the function of *Arabidopsis* bHLH TFs in multi aspects, including plant growth and development, stress response, biochemical functions and the web of signaling networks [36]. The genome-wide identification of bHLH TFs in *Aquilaria sinensis* illustrated that seven bHLH genes were involved in the wound-induced agarwood formation [37]. The genome-wide analyses of bHLH genes in *Cymbidium ensifolium* via bioinformatics techniques and molecular biology techniques showed that CebHLH13 and CebHLH75 played an important role in anthocyanin biosynthesis [38]. The identification of bHLH TFs in *Kandelia obovate* showed that 11 bHLH genes were related to the photoinhibition in photosystem II (PS II) under cold/drought stress [39]. The genome-wide identification and characterization of the bHLH genes in *Raphanus sativus* L. isolated four bHLH genes that participated in the photosynthesis process [40]. These results further indicated the importance of bHLH in various aspects of plants.

### Function of SlUPA-like in Multiple Hormone Pathways

Numerous studies have exhibited that bHLH transcription factors played a pivotal role in various aspects of plant growth and development through intricate protein–protein interactions with other key regulatory proteins. For instance, CgbHLH001 functioned positively in salt and drought stress tolerance via interacting with CgCDPK [41]. SlbHLH96 functioned as a positive regulator in drought tolerance by interacting with ERF4 and binding to the promoter of *CYP707A2* [42]. In cotton, GhDEL65 (bHLH TF) interacted with GhMYB2/3 (MYB TF) and GhTTG1 (WD40 protein), forming a ternary complex and having a positive effect on cotton fiber cell development [43]. SmbHLH60 interacted with SmMYC2 and participated in the regulation of phenolic acids and anthocyanin biosynthesis in *Salvia miltiorrhiza* [44]. Moreover, the evidence of the function of bHLH TFs in multiple plant hormones were also explored. For example, CES directly bound to the promoter of *GA2ox7* and participated in the regulation pathway of BR and GA [45]. bHLH113 integrated jasmonic acid/abscisic acid signaling and bound to the promoter of *DBR2 and ALDH1* and functioned as a positive regulator in the biosynthesis of artemisinin [46]. In the present study, SlUPA-like was used as bait to screen the interacted protein and the targeted interaction between SlUPA-like and SlMYB21 [47], SlMYC2 [48] and SlDELLA [49] were identified by Y2H and BiFC assay, illustrating that SlUPA-like possibly functioned in multiple hormone signal transduction pathways. SlMYB21, a MYB transcription factor in tomato, has been identified as a crucial regulator in the JA pathway. On the one hand, MYB21 functioned positively in JA biosynthesis and flower-to-fruit transition [47]. In addition, JA facilitated the transcript of *SlMYB21*, which coordinates flower opening, pollen maturation and gynoecium function in tomato [50]. On the other hand, *SlMYB21* was induced by exogenous GA in the *ga1-3 gai-t6 rga-t2 rgl1-1* quadruple mutant and was a crosstalk between gibberellin and jasmonate during the stamen development [51]. In previous studies, it has been demonstrated that DELLA protein GAI served as an inhibitor in the gibberellin signal transduction pathway [26]. It played a role not only in plant development [52] but also in abiotic stress [53] and plant hormone signal transduction [54]. Furthermore, DELLA was also employed in crosstalk between gibberellin and brassinosteroids [55,56]. The DELLA protein in *Arabidopsis* was able to repress the activation ability of *WRKY75*, which bound to the promoter of *FT* [57], attenuating the expression of its regulon. Tomato MYC2, a characterized bHLH transcription factor, served as a crucial controller in multiple aspects, such as plant morphogenesis [58], plant immunity [59] and abiotic stress [60], especially in the JA signaling pathway [61,62,63,64]. The overexpression of *BZR2* increased the content of JA/JA-ILE, and the interaction of MYC2 and BZR2 enhanced the activation of transcript levels of *GA20ox1L1* and *GA20ox2L2* mediated by MYC2, revealing the crosstalk of JA and BR [65]. Furthermore, MYC2 and MYB21 could form a bHLH-MYB complex and cooperatively regulate stamen development mediated by jasmonate [66]. Herein, in *SlUPA-like*-overexpressed lines, the transcript level of *SlBRI1* [67], *SlCPD*, *SlIBH1* [68] and *SlBZR1* [69] was induced, while *SlDWARF* [70] was inhibited significantly. Together with the increased GA, JA and BR content and the protein–protein interaction between SlUPA-like and MYB21, MYC2 and DELLA, we calculated that SlUPA-like possibly functioned at a cross talk between different plant hormone pathways.

Subsequently, we found that the transcript level of *JAZ2* (Solyc12g009220), *OPR1* (Solyc10g086220), *GID1-B* (Solyc06g008870) and *GID2* (Solyc04g078390), which functioned in the JA and GA signal transduction pathways, was enhanced significantly (Figure 4A,B), illustrating that the overexpression of *SlUPA-like* possibly altered the JA and gibberellin response pathways. Based on the promoter *cis*-acting analyses, we cloned the 2kb sequence and identified the regulation of *SlUPA-like* on *SlGID2* by employing Dual-LUC assay. Further, we proved that SlUPA-like activated *SlGID2* by binding to the E-box. As an F-box protein, *SlGID2* was essential for DELLA protein degradation mediated by GA. The interaction of DELLA and GID2 was disrupted when *N. benthamiana* was infected by ageratum leaf curl Sichuan virus (ALCScV), leading to a decreased content of GA [71]. In addition, GID2 has been identified as a positive controller and promoter of the gibberellin signal pathway in tomato by RNAi [72], illustrating its importance in the gibberellin pathway. In the present study, we cloned a 2kb promoter sequence and performed a Dual-LUC assay, obtaining evident upregulated results. These results further revealed the importance of SlUPA-like in plant hormone regulation.

Collectively, in present study, we overexpressed *SlUPA-like*, a typical bHLH transcription factor gene in the wild type, under the control of *35S* promoter. The resultant transgenic lines displayed discernible alterations in plant morphology, a suppression of photosynthesis and modifications in plant hormone levels. Furthermore, through Y2H and BiFC assays, we successfully identified protein–protein interactions between SlUPA-like and key regulators such as SlMYC2, SlMYB21 and SlDELLA, shedding light on its potential involvement in multiple hormone pathways. In addition, SlUPA-like could control the transcription of *SlGID2* by binding the E-box in its promoter, participating in the regulation of the gibberellin pathway. Our findings contribute valuable insights for a deeper understanding of the functional roles of *SlUPA-like* and its homologous genes in plant biology.

## 4. Materials and Method

### 4.1. Plant Materials and Growth Condition

In this study, the wild-type tomato, *Solanum lycopersicum* L. Ailsa Craig (AC^++^), was used as the wild-type experimental material and was grown in a greenhouse with a cycle of 16 h days (28 °C) and 8 h nights (18 °C), 0.25 mM × (m × s)^−2^. Thirty days after it was transplanted into soil, young leaf samples were collected, rapidly frozen in liquid nitrogen and stored at −80 °C for subsequent analyses. 

### 4.2. Protein Multiple Sequence Alignment and Cis-Acting Elements Identification

The protein sequences of homologous genes for *SlUPA-like* gene in other species, including *At* (*Arabidopsis thaliana*), *Os* (*Oryza sativa*), *Md* (*Malus domestica*), *Vv* (*Vitis vinifera*), *Nt* (*Nicotiana tabacum*), *Gm* (*Glycine max*), *Mt* (*Medicago truncatula*) and *Sb* (*Sorghum bicolor*), were identified and downloaded using the BlastP on the NCBI website. DNAMAN (V6, 0, 3, 99) was used to perform the protein multiple sequence alignment.

The promoter sequence of *SlUPA-like*, comprising 3000 bp upstream of the start codon ATG, was downloaded from the Sol Genomics Network (https://solgenomics.net/) (accessed on 9 August 2024). The *cis*-acting elements within this sequence were analyzed using online databases PlantCARE/Cis-acting regulatory element (https://bioinformatics.psb.ugent.be/webtools/plantcare/html/) (accessed on 12 September 2024).

### 4.3. The Extraction of Total RNA and Quantity Real Time PCR

The RNA extraction of leaf samples was performed using RNAiso Plus (Takara Bio Inc., Shiga, Japan) under the guidance of the instructions. The Oligd(T)_20_ and M-MLV reverse transcriptase were employed to synthesize the cDNA. Soon afterwards, the GoTaq qPCR master mix (Promega Corporation, Madison, WI, USA) was applied for qRT-PCR analysis with the 10 μL system, including 1.0 μL cDNA, 1.0 μL mixed primers, 3.0 μL ddH_2_O and 5.0 μL qMix enzyme, under the control of the CFX Connect real-time system (Bio-Rad, Hercules, CA, USA). Because of its expression stability in different tissues, *SlCAC* [73] (accession number: *SGN-U314153*) was used as the internal control when analyzing the expression of genes. The 2^−ΔΔCT^ method was employed to quantify the relative expression level [74]. All the primers are listed in Table S2.

### 4.4. The Determination of Hormone Content

50 mg of leaf sample was weighed in a 2 mL plastic microtube and frozen in liquid nitrogen, dissolved in 1 mL methanol/water/formic acid (15:4:1, *V*/*V*/*V*). 10 μL of internal standard mixed solution (100 ng/mL) was added into the extract as internal standard (IS) for the quantification. The mixture was vortexed for 10 min, then centrifugated for 5 min (12,000 r/min, and 4 °C); the supernatant was transferred to clean plastic microtubes, followed by evaporation to dryness, dissolution in 100 μL 80% methanol (*V*/*V*) and filtration through a 0.22 μm membrane filter for further analyses. The sample extracts were analyzed using an UPLC-ESI-MS/MS system (UPLC, ExionLC™ AD, Sciex, Framingham, MA, USA; MS, QTRAP^®^ 6500+, Sciex, Concord, ON, Canada). The analytical conditions were as follows, LC: column, Waters ACQUITY UPLC HSS T3 C18 (100 mm × 2.1 mm i.d., 1.8 µm); solvent system, water with 0.04% acetic acid (A), acetonitrile with 0.04% acetic acid (B); gradient program, started at 5% B (0–1 min), increased to 95% B (1–8 min), 95% B (8–9 min), finally ramped back to 5% B (9.1–12 min); flow rate, 0.35 mL/min; temperature, 40 °C; injection volume: 2 μL [75,76,77].

### 4.5. The Yeast Two-Hybrid Experiment

To identify the physical interaction of *SlUPA-like* with other proteins, the yeast two-hybrid assays were performed with the Clontech Matchmaker GAL4 system III. Based on the NCBI, the CDS (coding sequence) of *SlUPA-like* was isolated and cloned into pGBKT7 bait vector. Transactivation assay showed that *SlUPA-like* has a self-activation ability. To ensure the accuracy of the experiment, the coding sequence of *SlUPA-like* was inserted into pGADT7 vector, while the CDS sequence of *SlDELLA*, *SlPRE3*, *SlPRE4*, *SlMYB21* and *SlMYC2* were cloned into pGBKT7 vector. After sequencing verification, the combination of constructed pGADT7 and pGBKT7 were transformed into Y2H Gold yeast strain by the LiAc method, and transformants were grown on yeast media YPD (glucose medium) without Leu and Trp (SD/-Leu/-Trp plates). The PCR reaction was performed to validate the transformation. For testing the interaction accurately in yeast, the positive clone was cultured using the liquid culture SD/-Leu/-Trp medium and incubated at 30 °C until the culture reached OD_600_ = 0.5(mid-log phase); the culture was diluted (1, 1/10, 1/100), and 5 μL was patched on SD/-Ade/-His/-Leu/-Trp plates containing 5-bromo-4-chloro-3-indolyl-α-d-galactopyranoside (X-α-Gal) and incubated at 30 °C for 3 days. The primers used for this experiment are listed in Table S3.

### 4.6. The Bimolecular Fluorescent Complimentary (BiFC) Assay

The BiFC experiment was performed to further confirm the interaction results from the Y2H. The expression vector pFGC-GFP and confocal laser scanning microscope (Leica, Wetzlar, Germany) were used to verify the interaction. The full-length of the coding sequence of *SlUPA-like* without stop codon was cloned into the pFGC-nGFP to generate the fusion protein *SlUPA-like*-N with N-GFP while the CDS of candidate genes without stop codon were inserted into pFGC-cGFP to form the fusion protein with C-GFP, respectively. Then, the constructed vectors were transformed into *Agrobacterium tumefaciens* strain GV3101, while the infiltration of *Nicotiana benthamiana* was performed as described in Peng Y et al. [78]. The primers used for this experiment are listed in Table S4.

### 4.7. The High-Throughput Sequencing and Construction of cDNA Library 

Two biological replicates of leaves from the wild type and transgenic lines were employed to extract the nucleic acids. Total RNA was utilized as the starting material for RNA sample preparation. In brief, mRNAs with polyA tails were enriched using magnetic beads with Oligo(dT). The enriched RNA was then fragmented using a fragmentation buffer. Random N6 primers were used for reverse transcription, followed by second-strand synthesis to generate double-stranded DNA (dsDNA). The ends of the dsDNA were polished and phosphorylated at the 5′ ends, and an “A” overhang was added at the 3′ ends. Bubble-shaped adapters with a 3′ “T” overhang were ligated to the dsDNA. The ligated products were amplified by PCR using specific primers. The PCR products were denatured into single strands, and a bridge primer was used to circularize the single-stranded DNA to generate a single-stranded circular DNA library. The detailed information of reads were shown in Table S5. Sequencing was performed using the BGISEQ-500 platform. The original data were counted through the SOAPnuke (v1.4.0, Parameter settings: -l 5 -q 0.5 -n 0.1) filtering software to clean up the raw data with low quality, linker contamination and an excessive content of unknown bases N. It was aligned with the reference genome (ITAG3.2_ftp.solgenomics.net) through HISAT (v2.1.0, Parameter settings: --dta --phred64 unstranded --new-summary -x index -1 read_r1 -2 read_r2(PE)) [79], then aligned with the reference gene sequence using Bowtie2 (v2.2.5, Parameter settings: -q --phred64 --sensitive --dpad 0 --gbar 99,999,999 --mp 1,1 --np 1 --score-min L,0,-0.1 -p 16 -k 20) [80]; finally, the expression of known genes and new transcripts was calculated through RSEM (v1.2.8) [81].

### 4.8. The Analysis of DEGs (Differentially Expressed Genes)

In the present study, the transcript level of each gene was quantified in FPKM (Fragments Per Kilobase of exon model per Million mapped fragments) [82]. The DEGseq method and DESeq2 (1.20.0), based on the Poisson distribution, was used for detecting differentially expressed genes following the approach described by Wang et al. [83]. The criteria for screening differentially expressed genes were set as follows: Fold Change ≥ 2 and Adjusted *p* value ≤ 0.001.

### 4.9. The Analysis of GO Enrichment and KEGG Enrichment

For GO enrichment, the UniProt Database (http://www.uniprot.org/) (accessed on 10 October 2024) and whole protein sequence from genome of *Solanum lycopersicum* were used to obtain the GO annotation and then the UniProt ID were distributed into three basic GO categories, cellular component, biological process and molecular function. For KEGG enrichment, the KEGG GENES database with the KEGG automatic annotation server (KAAS) was operated for the acquisition of KEGG annotation. The results contained automatically generated KEGG pathways and KEGG orthology (KO) assignments [84]. 

### 4.10. Dual LUC Assay Procedure

The specific primers were designed to clone the CDS sequence of *SlUPA-like* and cloned into the multiple clone site on pGREEN II-62 SK vector. The 2 kb promoter sequence of candidate genes before start code ATG was obtained by PCR amplification from genome DNA and cloned into pGREEN II-0800 LUC. The finished vectors were confirmed by sequencing, and the completed vectors were transformed into *Agrobacterium tumefaciens* GV3101. After activation and expansion culture, the bacterial cells were collected and resuspended in the working solution. The OD_600_ and was adjusted and injected into leaves of tobacco. After three days, the leaves were collected and ground in liquid nitrogen. The cell lysates were added, and the supernatant collected after high-speed centrifugation. The RLU (relative light unit) was examined, and the ration of LUC/REN was calculated. All the primers are listed in Table S6.

### 4.11. EMSA Experiment

The full-length CDS of *SlUPA-like* was amplified and cloned to pGEX-4T-1, generating *GST-SlUPA-like* fusion expression vector. The validated vector was transformed by Sanger sequencing into BL21(DE3). The IPTG (Isopropyl-beta-D-thiogalactopyranoside) was employed to induce the expression of the fused protein, GST-*SlUPA-like*. After 12 h (with 0.2 mmol/L IPTG, 16 °C, 150 rpm), the bacterial cells were collected and subjected to ultrasonic fragmentation, releasing their contents and further purifying the GST-*SlUPA-like* fused protein. E-box motifs in the 2 kb promoter region were identified. Bio-P and Bio-mP were labeled by 5′-biotin, CP and mCP without biotin label. The mutant probes were converted by six bases in motif into A. The probes of different types and concentrations were co-incubated with the purified GST-*SlUPA-like* and then subjected to polyacrylamide gel electrophoresis. Subsequently, Western Blot was performed, and further analysis was performed by employing a Light Shift™ EMSA Optimization & Control Kit from Thermo Scientific (Waltham, MA, USA). All the primers are listed in Table S7.

### 4.12. Statistical Analysis and Heatmap Drawing

The data were obtained through repeated measurements. The significant differences among the means were calculated under the *t* test by Prism (8.0.2), developed by GraphPad Software Company (GraphPad Software, Inc., San Diego, CA, USA) in the United States. The drawing of the heatmap was conducted by TBtools v1.120 [85], which was developed by South China Agricultural University.

## 5. Conclusions

This study illustrated that the overexpression of *SlUPA-like*, a typical bHLH transcription factor gene, altered the endogenous GA, JA and BR content. qRT-PCR and RNA-Seq data also identified that the transcript level of genes involved in the biosynthesis, degradation and signaling transduction of these plant hormones were significantly influenced. Subsequently, the protein–protein interactions between *SlUPA-like* and *MYB21*, *MYC2* and *DELLA* were characterized by Y2H and BiFC. Moreover, Dual LUC and EMSA assays proved that *SlUPA-like* possessed the ability to bind to the promoter of *SlGID2* via E-box motif. In brief, these results illustrated that *SlUAP-like* possibly functioned as a crosstalk in multiplant hormones; the elucidated regulatory mechanism is expected to provide theoretical support for the molecular breeding of tomatoes.

## Data Availability

All relevant data are included within the article and its Supplemental Files.

## Supplementary Materials

The following supporting information can be downloaded at: https://www.mdpi.com/article/10.3390/ijms252413419/s1.
