# Supplementary material for: SlUPA-like, a bHLH Transcription Factor in Tomato (Solanum lycopersicum), Serves as the Crosstalk of GA, JA and BR
Source: Int J Mol Sci. 2024 Dec 14;25(24):13419. doi: 10.3390/ijms252413419 (PMC11677128; doi:10.3390/ijms252413419)
Supplement: Supplementary file 1 [file ijms-25-13419-s001.zip › ijms-3331999-supplementary.pdf]

Supplementary materials

Figure S1

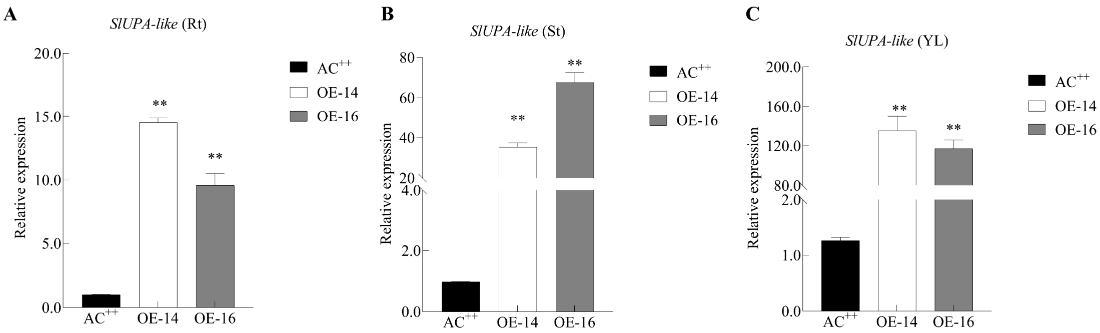

The examination of transcript level of *SlUPA-like* in different tissues, (A), Rt, root; (B), St, stem; (C), YL, young leaves. Error bars represented the standard error of the mean (n = 3). (**) $p < 0.01$ between the AC^{++} and transgenic plants by the t test.

Figure S2

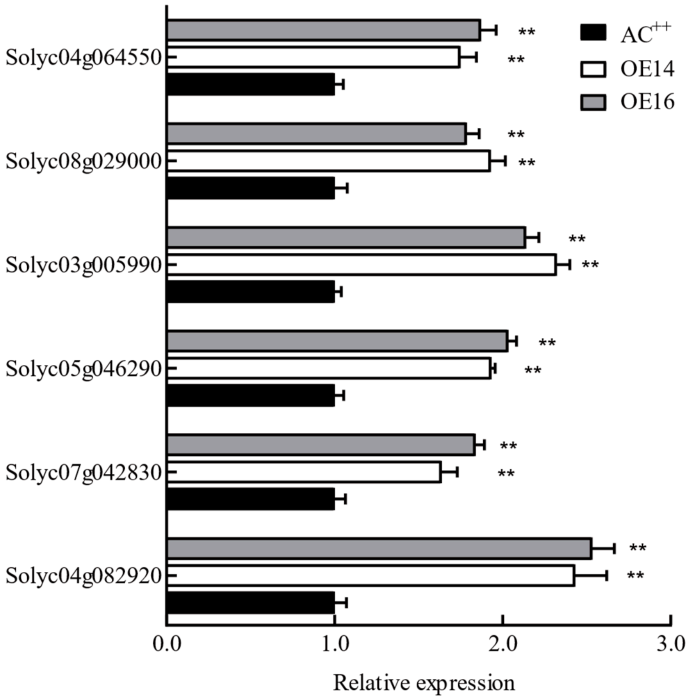

The validation of RNA-Seq by qRT-PCR.

Error bars represented the standard error of the mean (n = 3). (**) $p < 0.01$ between the AC^{++} and transgenic plants by the t test.

Figure S3

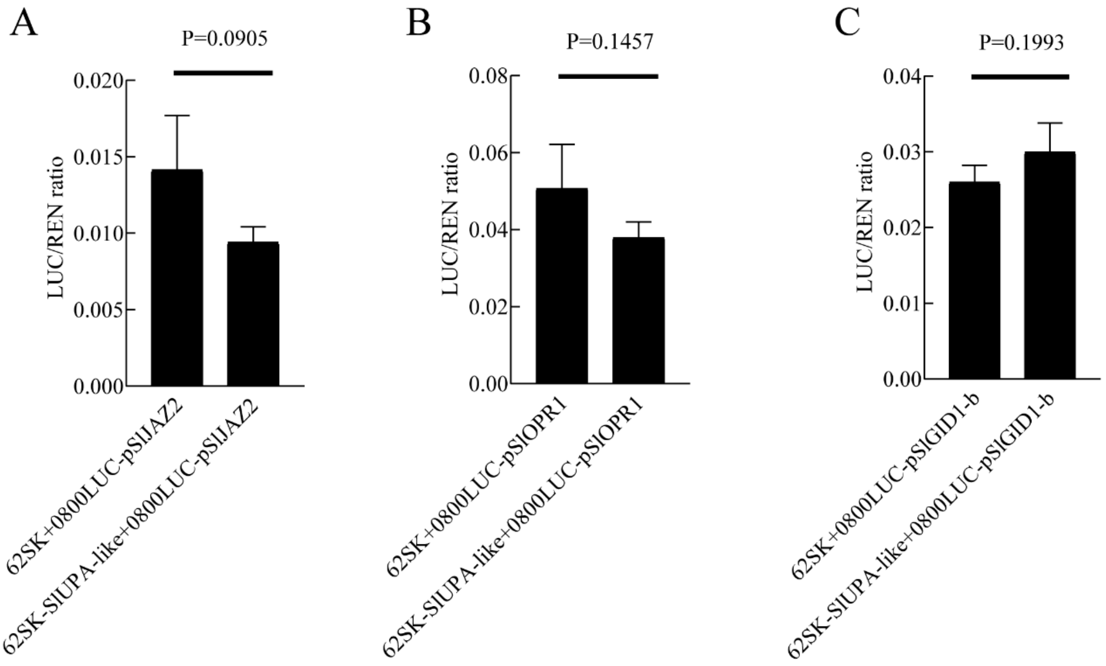

The Dual-LUC assay about SlUPA-like on *SlJAZ2*, *SlOPR1* and *SlGID1-b*.

Table S1 The prediction of *cis*-acting in promoter region

cis-acting	Function
ACGTG	abscisic acid responsiveness
GCCACT	meristem expression
CGTCA	MeJA-responsiveness
CCGAAA	low-temperature responsiveness
CAACTG	drought-inducibility
CATGCATG	seed-specific regulation
CCATCTTTTT	salicylic acid responsiveness
TATCCCA	gibberellin-responsiveness
CACGTT	light responsiveness

Table S2 Primers for qRT-PCR

Primer name	Sequence
qSlUPA-like	GGGTCTTCTTCTATGACTTCTGCT
	TTTCATCTTTGGCTACTAACTTGC
qSlCAC	CCTCCGTTGTGATGTAACTGG
	ATTGGTGGAAAGTAACATCATCG
qSlBRI1	GAGGTTTGGCTTTCCTACACC
	TCCGAGATCAGATACTCTGGCT
qSlBZR1	TCCTAAACACTGTGATAACAACGAG
	GGCTTACAGATGCTGAATAACCTA
qSlDWARF	GTGAATGAAGCGAAAGGACTG
	TCATGTACTTGTGAGCTGAACCA
qSlCYP734A7	TGGCAGCAATTAGACGAGCA
	GAGGCCAAAAGGCATGAACG
qIBH1	GCAGATATAGCAATGGCTTCAAC
	TTGTTTGTATCATCTTTTAAAGCATTT
qSlCPS	AGGTCTTGTTTTGGCTCCCC
	CAAGTAGTGATGGATGTCTCTGCC
qSlKAO	TGGACTTACACCAAAGGTAGGAA -
	AAATACATCACTGGACAAGACGG -
qSlGA20 ox1	TTCTCAAATTGGCTTCATGATCAA
	TTCCCCCTAATTCCCATAACAT
qSlGA20 ox2	TAAGAAGGATAAGGTGGTGAGGC
	CCGTAGTTTTCTGTTGAAGCCA
qSlGA2ox2	ATTAAGATCCAATAACACTTCG
	TCTTGATTTCACACTATTTGC
qSlGA2ox4	ATTCTTCTCCTCTCCCCTCTCTGA
	GACACATAATCATTCACCGCAGC
qSlGAST1	CAACAACAGAGAAATAACCAAC

	TTATACGATGTCTTTGAACACC
qSlGID2	GCGGTGTTGTTGAATGAGAATC
	GTCTTGTGCAGATCAGCTCCC
qSlGAI	CCAGCACTTGTCATTCTTACCC
	AAAGCTCATCCATTCCAGCA
qSlOPR3	TGCCTATCTTCATGTAACACAGCC
	AAAGTCCTCATTAAACGAGCCTCT
qSlLOXD	AGCAATAGCAGGAGTGAACCCA
	GCATCCAAAGCCTCTTGAACAG
qSlCOI1	CAACGATGGAAATAATGCAGAAAC
	TGGACAAGACACCTAAAAAGGAAG
qSlJA1	AGAAGAGCAAGATGGAAGACTAAGC
	GCATGATCCTTCTGTTTCCTTGT
qSlMYC2	GGGTTCTGGTTCATGGGC
	TTGCTGAGGAGGATTCTTCTGT
qSolyc05g046290	AATCCTCAAAGCATCATATTTTCAG
	CACCTCTTGTAGCCCAATCATCT
qSolyc04g082920	TGCCAAGCCCTTTTGATGG
	AGGCAAGTAAATTCCTAACGGTTC
qSolyc07g042830	ATGGATGCAGGTAAATGGAACAC
	CATAATGCTCCATGTTGTGAATAGTC
qSolyc03g005990	AAGATGGAACTACTTACAAAAAGGGA
	ATGAGCAGGGGCTTACAGATG
qSolyc08g029000	ATTCCCTCCAAAAAGCGAGTTA
	TCGATCGTTAGCCCATCTAGTGTA
qSolyc04g064550	TTCAAGGGATAGCCAAGAGAGG
	CCACTTCCCTGCAACCTCATA

Table S3 Primers for Y2H assay

Primer ID	Sequence (5’-3’)
Y2H-UPA-F	GGAATTCCATATGATGGCTGCTTTTTCATCACACC
Y2H-UPA-R	CGGGATCCTTAATGGAAAGAACAAAAGTTGTTGC
Y2H-GAI-F	GGGAATTCCATATGATGAAGAGAGATCGAGATCGAGATC
Y2H-GAI-R	CGCGGATCCTTACAACTCGACTTCTCCGGC
Y2H-MYC2-F	GGGAATTCCATATGATGACTGAATACAGCTTGCCCAC
Y2H-MYC2-R	CGCGGATCCTTAGTGTGTTTCAGCAATTTTCGAT
Y2H-MYB21-F	GGGAATTCCATATGATGGATAAAATATGCAACTCTCAAGAT
Y2H-MYB21-R	CGCGGATCCTTAATCTCCATTAAGCAATTGCATT
Y2H-PRE3-F	CCGGAATTCATGTCTAGTAGAAGGTCAAGATCATCA
Y2H-PRE3-R	CGCGGATCCCTATTGCATAAGTAGGCTTCTAATTAGA
Y2H-PRE4-F	CCGGAATTC ATGTCAAGCAGACGATCACGTT
Y2H-PRE4-R	CGCGGATCCCTACATAAGTAAGCTTCTAATAATAGCAGCT

Table S4 Primers for BiFC assay

Primer ID	Sequence
BiFC -UPA-like-F	CGAGCTCATGGCTGCTTTTTCATCACACC
BiFC -UPA-like-R	CGCGGATCCATGGAAAGAACAAAAGTTGTTGCTAA
BiFC -DELLA-F	CGAGCTCATGAAGAGAGATCGAGATCGAGATC
BiFC -DELLA-R	CGCGGATCCCAACTCGACTTCTCCGGCG
BiFC -MYC2-F	CGAGCTCATGACTGAATACAGCTTGCCCAC
BiFC -MYC2-R	CGCGGATCCGTGTGTTTCAGCAATTTTCGATGT
BiFC -MYB21-F	CGAGCTCATGGATAAAATATGCAACTCTCAAGATG
BiFC -MYB21-R	CGCGGATCCATCTCCATTAAGCAATTGCATTGAC

Table S5 Transcriptome information

Sample	Total Raw Reads (Million)	Total Clean Reads (Million)	Total Clean Bases (Gb)
OE14	47.33	43.1	6.47
OE16	47.33	44.2	6.63
AC^{++}-1	49.08	43.63	6.54
AC^{++}-2	47.33	43.52	6.53

Table S6 Primers for Dual-LUC assay

Primer ID	Sequence
62SK-UPA-F	aggacagcccaagctgagctcATGGCTGCTTTTTCATCACACC
62SK-UPA-R	gtcgacggtatcgataagcttTTAATGGAAAGAACAAAAGTTGTTGC
LUC-pGID2-F	ctatagggcgaattgggtaccTGATTTACTGTATTATTTTTTTCGTCTTAAA
LUC-pGID2-R	atcgataccgtcgacctcgagCGTTGGATCTAAATTTGTGAAAATTG
LUC-pOPR1-F	ctatagggcgaattgggtaccAGATAAAGTAAAGGATTTGGTTGAAATT
LUC-pOPR1-R	atcgataccgtcgacctcgagTGGTGTAAGCTTTCTTGTTAGTGATTAT
LUC-pJAZ2-F	ctatagggcgaattgggtaccACTCTATTAAATTATTTAAATACAAGAAATTACATT
LUC-pJAZ2-R	atcgataccgtcgacctcgagCTTGTTTTTCCGGCCAAAAA
LUC-pGID1-B-F	ctatagggcgaattgggtaccGAGAAGAAAGTGGAAGTCACTTGTACTT
LUC-pGID1-B-R	atcgataccgtcgacctcgagGAAAGAAAGTAAAATTTACAGCTTAAAATAGA

Table S7 Primers for EMSA assay

Primer ID	Sequence	Tag
UPA-like-pGEX4T-1-F	GATCTGGTTCCGCGTGGATCCATGGCTGCTTTTTCATCACACC	
UPA-like-pGEX4T-1-R	GTCACGATGCGGCCGCTCGAGTTAATGGAAAGAACAAAAGTTGTTGC	
Motif-1BP-F	ATTAACAGGCAGTAACAATTCAGTTGAAAGATTTTTGAAGAATATTTTAG	5`Biotin
Motif-1BP-R	CTAAAATATTCTTCAAAAATCTTTCAACTGAATTGTTACTGCCTGTTAAT	5`Biotin
Motif-1mBP-F	ATTAACAGGCAGTAACAATTAAAAAAAAAGATTTTTGAAGAATATTTTAG	5`Biotin
Motif-1mBP-R	CTAAAATATTCTTCAAAAATCTTTTTTTTTAATTGTTACTGCCTGTTAAT	5`Biotin
Motif-1CP-F	ATTAACAGGCAGTAACAATTCAGTTGAAAGATTTTTGAAGAATATTTTAG	
Motif-1CP-R	CTAAAATATTCTTCAAAAATCTTTCAACTGAATTGTTACTGCCTGTTAAT	
Motif-1mCP-F	ATTAACAGGCAGTAACAATTAAAAAAAAAGATTTTTGAAGAATATTTTAG	
Motif-1mCP-R	CTAAAATATTCTTCAAAAATCTTTTTTTTTAATTGTTACTGCCTGTTAAT	
Motif-2BP-F	AAGTTAAAAGCCAAAGTTATCAACTGACGTCAATTTAAAGGTCATGTTTA	5`Biotin
Motif-2BP-R	TAAACATGACCTTTAAATTGACGTCAGTTGATAACTTTGGCTTTTAACTT	5`Biotin
Motif-2mBP-F	AAGTTAAAAGCCAAAGTTATAAAAAAACGTCAATTTAAAGGTCATGTTTA	5`Biotin
Motif-2mBP-R	TAAACATGACCTTTAAATTGACGTTTTTTTATAACTTTGGCTTTTAACTT	5`Biotin
Motif-2CP-F	AAGTTAAAAGCCAAAGTTATCAACTGACGTCAATTTAAAGGTCATGTTTA	
Motif-2CP-R	TAAACATGACCTTTAAATTGACGTCAGTTGATAACTTTGGCTTTTAACTT	
Motif-2mCP-F	AAGTTAAAAGCCAAAGTTATAAAAAAACGTCAATTTAAAGGTCATGTTTA	
Motif-2mCP-R	TAAACATGACCTTTAAATTGACGTTTTTTTATAACTTTGGCTTTTAACTT	
